# Total bilirubin-to-albumin ratio and short- and long-term all-cause mortality in acute pancreatitis: Evidence from the MIMIC-IV database

**DOI:** 10.1371/journal.pone.0323330

**Published:** 2025-05-22

**Authors:** XingYi Yang, Min Zhang, LiHong Lv, XuYong Chen, ZhenMei Li

**Affiliations:** Department of Gastroenterology Disease, XianJu People’s Hospital, Zhejiang Southeast Campus of Zhejiang Provincial People’s Hospital, Affiliated Xianju’s Hospital, Hangzhou Medical College, Xianju, Taizhou, Zhejiang, China; Rutgers: Rutgers The State University of New Jersey, UNITED STATES OF AMERICA

## Abstract

**Background:**

The Total Bilirubin-to-Albumin Ratio (TBAR) is widely recognized and applied as a biomarker in the prognostic evaluation of various diseases. However, its role in predicting survival outcomes in patients with acute pancreatitis (AP) remains underexplored. This study aims to investigate the association between TBAR levels and mortality rates in AP patients, thereby providing a novel prognostic indicator for clinical use.

**Methods:**

This study investigates the association between TBAR and mortality in AP patients. We stratified patient data using X-tile software to analyze intergroup differences. Risk factors significantly associated with mortality were identified through univariate and multivariate regression analyses. Kaplan-Meier (KM) analysis evaluated TBAR’s impact on survival, while Receiver Operating Characteristic (ROC) analysis assessed its predictive accuracy, sensitivity, and Area Under the Curve (AUC) for mortality. To ensure robustness, we used Restricted Cubic Spline (RCS) modeling to explore non-linear relationships and performed subgroup analyses to verify the consistency of the TBAR mortality association across patient subgroups.

**Result:**

This study included 477 patients. Using X-tile software, we set the optimal TBAR cutoff at 1.33 based on 28-day mortality. Patients were categorized into high-risk (TBAR ≥ 1.33) and low-risk (TBAR < 1.33) groups. Elevated TBAR significantly correlated with increased mortality at multiple time points (7, 14, 21, 28, 90, and 365 days; P < 0.05). KM analysis confirmed lower survival rates in the high-risk group at all time points (P < 0.05). ROC analysis showed TBAR’s predictive accuracy for mortality was comparable to the SOFA score and superior to other indicators. RCS modeling revealed a linear TBAR mortality relationship. Subgroup analyses showed no significant interactions between TBAR and most subgroups.

**Conclusion:**

The TBAR is strongly correlated with short-term and long-term mortality in patients with acute pancreatitis.

## 1 Introduction

Acute pancreatitis (AP) is a common acute emergency of the digestive system, with a rising incidence worldwide. The global annual incidence of AP ranges from 13 to 45 cases per 100,000 individuals, and recent increases have been attributed to changes in lifestyle and dietary habits [1]. The pathogenesis of AP primarily involves injury to pancreatic acinar cells and the activation of inflammatory responses. Normally, digestive enzymes secreted by pancreatic acinar cells exist in an inactive form. However, in AP, these enzymes are abnormally activated within the pancreas, leading to autodigestion of pancreatic tissue and subsequent inflammation [2]. This inflammatory activation further triggers the release of inflammatory mediators, such as tumor necrosis factor-α (TNF-α) and interleukin-6 (IL-6), which exacerbate local pancreatic inflammation and may induce systemic inflammatory response syndrome (SIRS) and multiple organ dysfunction syndrome (MODS) [3]. Mortality rates in severe AP can reach 20–30% [4]. Therefore, early identification and assessment of prognostic risks in patients with AP are crucial for improving survival rates.

In the prognostic evaluation of AP, serum biomarkers have increasingly attracted attention. Total bilirubin (TB) and albumin (ALB) are two widely used biochemical indicators, and their prognostic significance in AP has been extensively demonstrated. Total bilirubin is a key indicator of liver function, with elevated levels often reflecting liver dysfunction or biliary obstruction [5]. Elevated serum total bilirubin levels are closely associated with disease severity and poor prognosis in AP [6]. Albumin, an essential plasma protein, is typically reduced in the presence of inflammatory responses, malnutrition, and liver dysfunction [7]. Hypoalbuminemia is an established independent risk factor for poor prognosis in patients with AP [8].

The Total Bilirubin-to-Albumin Ratio (TBAR) is a novel prognostic indicator that integrates the prognostic information of total bilirubin and albumin, potentially offering a more comprehensive reflection of systemic inflammation and liver function. Previous studies have demonstrated the potential value of the indirect bilirubin-to-albumin ratio in predicting mortality among patients with hepatic encephalopathy [9]. However, the prognostic significance of TBAR in AP remains to be elucidated. This study aims to leverage the MIMIC-IV database [10] to investigate the association between TBAR and both short-term and long-term all-cause mortality in patients with AP, thereby providing a novel prognostic tool for clinical use.

## 2 Materials and methods

### 2.1 Database introduction

Data for this study were sourced from the MIMIC-IV (v2.2) database, a publicly accessible and comprehensive dataset developed by the Laboratory of Computational Physiology at the Massachusetts Institute of Technology. This database includes detailed records of over 70,000 ICU patients admitted to the Beth Israel Deaconess Medical Center (BIDMC) from 2012 to 2019. It encompasses a wide array of data types, including laboratory results, medication schedules, vital signs, and hospital stay durations. This database was approved by the Institutional Review Board of the Beth Israel Denconess Medical center (2001P-001699/14). All personal identifiers have been de-identified, with real patient data replaced by randomly assigned numbers. Consequently, the need for ethical approval and informed consent was waived.

### 2.2 Inclusion and exclusion criteria

In this investigation, we enrolled 5,894 patients with a diagnosis of AP, of whom 1,276 were admitted to the ICU. We applied the following exclusion criteria: (1) not the initial ICU admission; (2) ICU stay shorter than 24 hours; (3) absence of total bilirubin or albumin data within the first 24 hours of admission. After applying these criteria, a final cohort of 477 patients was selected for analysis.

### 2.3 Data collection

This study systematically collected multidimensional data from the database, including demographic characteristics, clinical vital signs, laboratory test indicators, comorbidities, and treatment outcomes. Specifically, vital signs data included key indicators such as blood pressure, heart rate, respiratory rate, and body temperature. In terms of laboratory parameters, we comprehensively recorded multiple indicators, including white blood cell count (WBC), red blood cells, platelet count, electrolytes (sodium, potassium), renal function indicators (blood urea nitrogen [BUN], creatinine), liver function indicators (bilirubin, albumin, aspartate aminotransferase [AST]; alanine aminotransferase [ALT]), coagulation function indicators (activated partial thromboplastin time [APTT], international normalized ratio [INR]), and lactic acid levels. Additionally, the study included clinical assessment data such as the Glasgow Coma Scale (GCS) [11] and Sequential Organ Failure Assessment (SOFA) [12] scores.

The TBAR was specifically selected as the core study variable. The study model was constructed based on the average values of parameters recorded within the first 24 hours of ICU admission for all patients. The study cohort strictly adhered to the diagnostic criteria for AP and followed up with patients for at least 365 days to assess their long-term prognosis. Table 1 provides a detailed list of the variables extracted in the study.

**Table 1:** Patient demographics and baseline characteristics.

Variables	Total	TBAR		p-value
		<1.33, N = 385^1^	≥1.33, N = 92^1^	
**Demographic**				
Age(year)	58 (46, 71)	58 (46, 72)	57 (45, 70)	0.448
Gender, n(%)				0.010
Female	213 (44.7%)	183 (47.5%)	30 (32.6%)	
Male	264 (55.3%)	202 (52.5%)	62 (67.4%)	
Race, n(%)				0.460
Other	181 (37.9%)	143 (37.1%)	38 (41.3%)	
White	296 (62.1%)	242 (62.9%)	54 (58.7%)	
**Vital Signs**				
Heart rate (beats/min)	101 (86, 117)	102 (87, 117)	100 (85, 113)	0.224
SBP (mmHg)	127 (110, 145)	129 (112, 147)	116 (101, 139)	0.003
DBP (mmHg)	73 (60, 85)	74 (61, 86)	66 (56, 83)	0.023
MAP (mmHg)	86 (73, 99)	87 (75, 100)	78 (69, 95)	0.002
Respiratory rate (beats/min)	21 (18, 25)	21 (18, 25)	20 (16, 25)	0.202
Temperature(°C)	36.83 (36.44, 37.33)	36.89 (36.50, 37.33)	36.75 (36.28, 37.07)	0.013
**Comorbidities**				
AKI, n(%)	357 (74.8%)	276 (71.7%)	81 (88.0%)	0.001
Sepsis, n(%)	330 (69.2%)	250 (64.9%)	80 (87.0%)	<0.001
Myocardial infarct, n(%)	47 (9.9%)	65 (16.9%)	12 (13.0%)	0.678
Congestive heart failure, n(%)	77 (16.1%)	65 (16.9%)	12 (13.0%)	0.368
Cerebrovascular disease, n(%)	27 (5.7%)	21 (5.5%)	6 (6.5%)	0.691
Chronic pulmonary disease, n(%)	105 (22.0%)	87 (22.6%)	18 (19.6%)	0.528
Peptic ulcer disease, n(%)	26 (5.5%)	18 (4.7%)	8 (8.7%)	0.127
Diabetes, n(%)	136 (28.5%)	113 (29.4%)	23 (25.0%)	0.406
Renal disease, n(%)	71 (14.9%)	59 (15.3%)	12 (13.0%)	0.581
Malignant cancer, n(%)	39 (8.2%)	28 (7.3%)	11 (12.0%)	0.141
Severe liver disease, n(%)	69 (14.5%)	32 (8.3%)	37 (40.2%)	<0.001
pancreatic necrosis, n(%)	9 (1.9%)	9 (2.3%)	0 (0.0%)	0.217
**Laboratory Indicators**				
TCa(mg/dl)	7.90 (7.30, 8.50)	7.90 (7.20, 8.50)	7.90 (7.40, 8.63)	0.231
Bun(mg/dl)	21 (13, 38)	20 (12, 35)	24 (15, 48)	0.030
Potassium (mEq/l)	4.10 (3.60, 4.50)	4.00 (3.60, 4.50)	4.10 (3.90, 4.60)	0.218
Sodium (mEq/l)	138.0 (135.0, 142.0)	139.0 (136.0, 142.0)	137.0 (132.0, 139.0)	<0.001
Glucose (mg/dl)	127 (103, 181)	133 (105, 189)	115 (95, 151)	<0.001
Creatinine (mg/dl)	1.10 (0.80, 2.00)	1.00 (0.70, 1.80)	1.40 (0.80, 2.70)	0.006
Total bilirubin (mg/dl)	1.1 (0.6, 3.0)	0.8 (0.5, 1.7)	7.1 (5.0, 12.5)	<0.001
AST(IU/L)	81 (39, 203)	63 (34, 157)	169 (102, 333)	<0.001
ALT(IU/L)	56 (26, 167)	47 (24, 135)	106 (53, 233)	<0.001
WBC(10^9/L)	12 (9, 18)	13 (9, 18)	12 (7, 19)	0.648
RDW(%)	14.60 (13.78, 15.90)	14.50 (13.60, 15.70)	15.60 (14.30, 17.73)	<0.001
RBC(10^9/L)	3.64 (3.15, 4.22)	3.70 (3.23, 4.29)	3.40 (2.81, 3.98)	<0.001
Platelet(10^9/L)	187 (127, 270)	199 (140, 285)	134 (84, 198)	<0.001
Albumin(mg/dl)	3.00 ± 0.63	3.01 ± 0.64	2.81 ± 0.59	0.479
Lactate(mg/dl)	1.90 (1.30, 3.20)	1.70 (1.20, 3.00)	2.30 (1.60, 3.90)	<0.001
INR	1.30 (1.20, 1.60)	1.30 (1.10, 1.43)	1.60 (1.30, 2.23)	<0.001
APPT(s)	31 (27, 39)	30 (26, 36)	36 (31, 47)	<0.001
Chloride(mEq/l)	104 (100, 109)	105 (101, 110)	102 (97, 105)	<0.001
Anion Gap(mEq/l)	16.0 (13.0, 19.0)	15.5 (13.0, 19.0)	17.0 (14.0, 21.3)	0.002
SOFA	1.00 (0.00, 3.00)	1.00 (0.00, 3.00)	3.00 (0.75, 7.00)	<0.001
GCS	15.00 (15.00, 15.00)	15.00 (15.00, 15.00)	15.00 (15.00, 15.00)	0.395
**Treatment**				
CRRT, n(%)	67 (14.0%)	42 (10.9%)	25 (27.2%)	<0.001
MV, n(%)	396 (83.0%)	317 (82.3%)	79 (85.9%)	0.418
ERCP, n(%)	27 (5.7%)	15 (3.9%)	12 (13.0%)	<0.001
**a**ntibiotics, n(%)	377 (79.0%)	293 (76.1%)	84 (91.3%)	0.001
Statins, n(%)	52 (10.9%)	42 (10.9%)	10 (10.9%)	0.991
Octreotide, n(%)	37 (7.8%)	18 (4.7%)	19 (20.7%)	<0.001
Fibrates, n(%)	56 (11.7%)	52 (13.5%)	4 (4.3%)	0.014
Vasopressin, n(%)	83 (17.4%)	57 (14.8%)	26 (28.3%)	0.002
**Clinical outcome**				
Hospital mortality	70 (14.7%)	42 (10.9%)	28 (30.4%)	<0.001
ICU mortality	48 (10.1%)	30 (7.8%)	18 (19.6%)	<0.001
7d mortality	24 (5.0%)	11 (2.9%)	13 (14.1%)	<0.001
14d mortality	42 (8.8%)	22 (5.7%)	20 (21.7%)	<0.001
21d mortality	54 (11.3%)	30 (7.8%)	24 (26.1%)	<0.001
28d mortality	63 (13.2%)	37 (9.6%)	26 (28.3%)	<0.001
90d mortality	96 (20.1%)	65 (16.9%)	31 (33.7%)	<0.001
365d mortality	118 (24.7%)	81 (21.0%)	37 (40.2%)	<0.001

SBP, systolic blood pressure; DBP, diastolic blood pressure; MAP, mean arterial pressure; CRRT, continuous renal replacement therapy; MV, mechanical ventilation; ERCP, endoscopic retrograde cholangiopancreatography; AKI, acute kidney injury; TCa, serum total calcium; BUN, Blood Urea Nitrogen; AST, aspartate aminotransferase; ALT, alanine aminotransferase; WBC, white blood cell; RDW, erythrocyte distribution width; RBC, red blood cell; INR, international normalized ratio; APPT, Activated Partial Thromboplastin Time; TBAR, Total.Bilirubin to Albumin Ratio; SOFA Sepsis-Related Organ Failure Assessment Score; GCS Glasgow Coma Scale;

The primary endpoint of this study was all-cause mortality at 7, 14, 21, 28, 90, and 365 days following hospital admission. During this period, we also evaluated ICU mortality and in-hospital mortality to comprehensively understand the short-term and long-term prognosis of patients.

### 2.4 Statistical analyses

This study employed a range of statistical methods for data analysis and presentation. Continuous variables were described based on their distribution characteristics: those conforming to a normal distribution were presented as mean ± standard deviation (Mean±SD), while non-normally distributed variables were described using the median (interquartile range, IQR). Categorical variables were reported as frequencies and percentages (n,%). In the analysis of baseline characteristics, statistical tests were selected according to the variable type: continuous variables were analyzed using the independent samples t-test or One-Way ANOVA, while categorical variables were assessed using Pearson’s chi-square test or Fisher’s exact test. Data with missing values exceeding 20% were deleted, while data with missing values less than 20% were subjected to random forest imputation. The study utilized X-tile software (Version 3.6.1, Yale University, USA) to stratify TBAR scores into two groups based on 28-day mortality outcomes after admission: TBAR<1.33 and TBAR≥1.33. Cox proportional hazards regression models were employed for both univariate and multivariate analyses to identify independent prognostic factors for mortality in AP patients at 7, 14, 21, 28, 90, and 365 days after admission. Hazard ratios (HRs) and their 95% confidence intervals (CIs) were calculated. K-M survival curves were plotted, and the log-rank test was used to compare survival differences between the two groups. To evaluate the predictive power of various indicators, ROC curve analysis was conducted, focusing on the predictive ability of TBAR, total bilirubin, albumin, Sequential Organ Failure Assessment (SOFA) score, and Glasgow Coma Score (GCS) for mortality at different time points (7, 14, 21, 28, 90, and 365 days). Sensitivity, specificity, and the AUC were calculated for each indicator. Additionally, RCS analysis was used to explore the potential linear relationship between TBAR and mortality. Subgroup analyses were performed to further validate the clinical application value of the TBAR score, assessing the impact of parameters such as age, sex, race, acute kidney injury (AKI), sepsis, and severe liver disease on the TBAR score. All statistical analyses were conducted using R software (Version 4.22) and MSTATA software.

## 3 Results

### 3.1 Characteristics of patients at baseline

**Fig 1:**
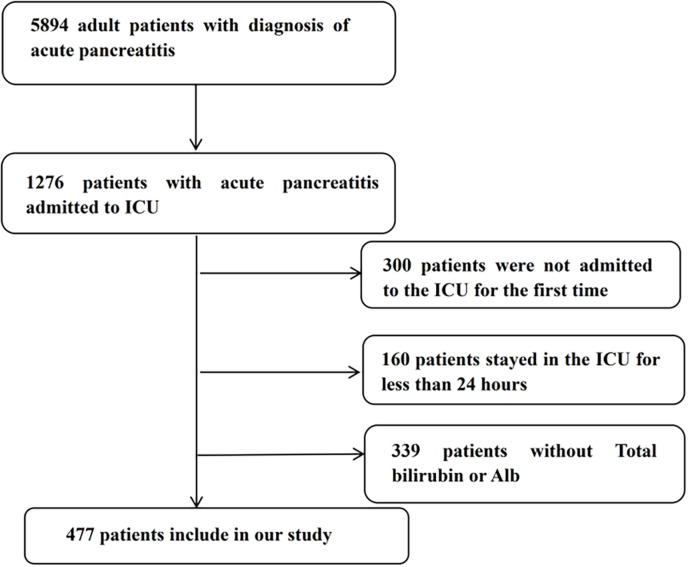
A flow diagram of study participants.

As shown in **Fig 1**, This study included 477 patients. Using X-tile software, we analyzed 28-day mortality and determined an optimal TBAR threshold of 1.33. Patients were categorized into two groups based on this threshold: the high-risk group (TBAR≥1.33) and the low-risk group (TBAR<1.33). The high-risk group had a mean age of 57 years, with 67.4% being female. This group exhibited significantly higher levels of several biochemical parameters, including blood urea nitrogen (BUN), creatinine (Cr), total bilirubin, alanine aminotransferase (ALT), aspartate aminotransferase (AST), red blood cell count (RBC), international normalized ratio (INR), lactate, activated partial thromboplastin time (APTT), anion gap, and SOFA score. Conversely, patients in the high-risk group had lower levels of sodium, chloride, glucose, hemoglobin, blood pressure, and body temperature compared to those in the low-risk group. Additionally, the incidence of complications such as AKI, sepsis, and severe liver disease was significantly higher in the high-risk group.

In terms of clinical outcomes, the ICU mortality rate was significantly higher in the high-risk group than in the low-risk group (30.4% vs. 10.9%, P < 0.001). Similarly, in-hospital mortality was significantly higher in the high-risk group (19.6% vs. 7.8%, P < 0.001). The high-risk group also had significantly higher mortality rates at multiple time points: 7 days (14.1% vs. 2.9%, P < 0.001), 14 days (21.7% vs. 5.7%, P < 0.001), 21 days (26.1% vs. 7.8%, P < 0.001), 28 days (28.3% vs. 9.6%, P < 0.001), 90 days (33.7% vs. 16.9%, P < 0.001), and 365 days (40.2% vs. 21.0%, P < 0.001). Detailed baseline characteristics and differences between the two groups are provided in **Table 1**.

### 3.2 Cox regression analysis of TBAR and mortality in AP patients

Cox regression analysis demonstrated a significant association between TBAR levels ≥1.33 and both short-term and long-term mortality in patients with AP, without adjustment for other variables. The HR and 95%CI were as follows: 7 days (HR = 4.07, 95%CI: 1.76–9.43, P = 0.001), 14 days (HR = 4.17, 95%CI: 2.28–7.64, P < 0.001), 21 days (HR = 3.76, 95%CI: 2.20–6.43, P < 0.001), 28 days (HR = 3.35, 95%CI: 2.03–5.53, P < 0.001), 90 days (HR = 2.35, 95%CI: 1.53–3.61, P < 0.001), and 365 days (HR = 2.29, 95%CI: 1.55–3.38, P < 0.001).

In Model 1, after adjusting for age and sex, the group with TBAR≥1.33 continued to exhibit significantly higher mortality at each time point: 7 days (HR = 5.67, 95%CI: 2.50–12.9, P < 0.001), 14 days (HR = 4.78, 95%CI: 2.57–8.86, P < 0.001), 21 days (HR = 4.18, 95%CI: 2.42–7.22, P < 0.001), 28 days (HR = 3.66, 95%CI: 2.20–6.10, P < 0.001), 90 days (HR = 2.63, 95%CI: 1.70–4.06, P < 0.001), and 365 days (HR = 2.48, 95%CI: 1.67–3.68, P < 0.001).

In Model 2, after further adjustments, the group with TBAR≥1.33 maintained significantly higher mortality rates, indicating an independent association between TBAR≥1.33 and mortality. The HR values were: 7 days (HR = 4.62, 95%CI: 1.74–12.3, P = 0.002), 14 days (HR = 3.15, 95%CI: 1.57–6.34, P = 0.001), 21 days (HR = 2.79, 95%CI: 1.52–5.13, P < 0.001), 28 days (HR = 2.27, 95%CI: 1.29–4.01, P = 0.005), 90 days (HR = 1.75, 95%CI: 1.09–2.82, P = 0.021), and 365 days (HR = 1.82, 95%CI: 1.18–2.80, P = 0.007). For detailed results, refer to **Table 2**.

**Table 2:** Association between TBAR and mortality in patients with AP.

Outcome	Unadjusted HR (95%CI)	Model1 HR (95%CI)	Model2 HR (95%CI)
**7-d**			
TBAR＜1.33	1	1	1
TBAR≥1.33	5.18(2.32, 11.6)	5.67(2.50, 12.9)	4.39(1.71, 11.3)
P	<0.001	<0.001	0.002
**14-d**			
TBAR＜1.33	1	1	1
TBAR≥1.33	4.17(2.28, 7.64)	4.78(2.57, 8.86)	3.58(1.78, 7.20)
P	<0.001	<0.001	<0.001
**21-d**			
TBAR＜1.33	1	1	1
TBAR≥1.33	3.76(2.20, 6.43)	4.18(2.42, 7.22)	3.50(1.90, 6.43)
P	<0.001	<0.001	<0.001
**28-d**			
TBAR＜1.33	1	1	1
TBAR≥1.33	3.35(2.03, 5.53)	3.66(2.20, 6.10)	2.93(1.66, 5.15)
P	<0.001	<0.001	<0.001
**90-d**			
TBAR＜1.33	1	1	1
TBAR≥1.33	2.35(1.53, 3.61)	2.63(1.70, 4.06)	2.17(1.35, 3.50)
P	<0.001	<0.001	0.001
**365-d**			
TBAR＜1.33	1	1	1
TBAR≥1.33	2.29(1.55, 3.38)	2.48(1.67, 3.68)	2.27(1.47, 3.50)
P	<0.001	<0.001	<0.001

Model 1: adjusted Age and gender.

Model 2: adjusted for Age, Gender, temperature, INR, platelet, AKI, Sepsis, Pancreatic necrosis, ERCP and Antibiotics.

### 3.3 Analysis of Kaplan Meier and ROC CURVES

**Fig 2:**
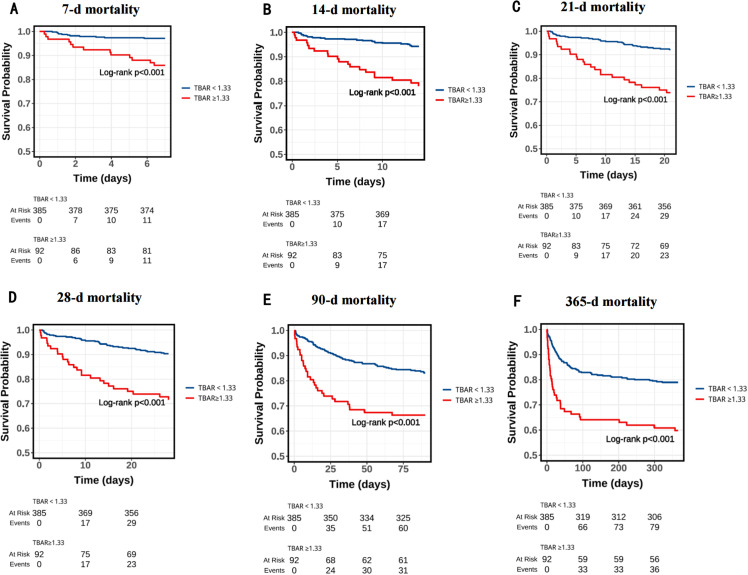
Kaplan-Meier survival analysis curves for all-cause mortality in patients with AP at 7-d (A), 14-d (B), 21-d (C), 28-d (D), 90-d (E), and 365-d (F) of hospital admission.

KM survival analysis showed that patients with TBAR≥1.33 had significantly higher mortality rates at multiple time points compared to those with lower TBAR values. The specific mortality rates were as follows: 7 days (11.9% vs. 2.8%, P < 0.001), 14 days (18.4% vs. 4.4%, P < 0.001), 21 days (25.0% vs. 7.5%, P < 0.001), 28 days (25.0% vs. 7.5%, P < 0.001), 90 days (33.6% vs. 15.5%, P < 0.001), and 365 days (39.1% vs. 20.5%, P < 0.001). These findings are illustrated in **Fig 2**.

**Fig 3:**
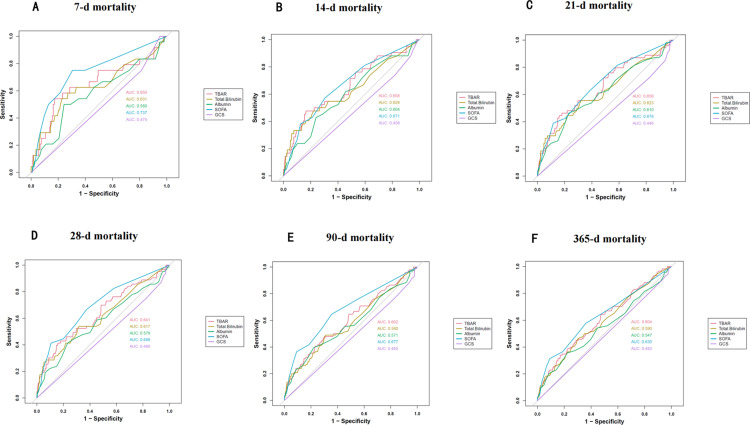
ROC curves for predicting all-cause mortality in patients with AP at 7-d (A), 14-d (B), 21-d (C), 28-d (D), 90-d (E), and 365-d (F) after admission.

Additionally, ROC curve analysis was used to evaluate the predictive ability of five parameters for all-cause mortality at different post-admission intervals: total bilirubin, albumin, TBAR, SOFA score, and GCS. The results indicated that the AUC for TBAR was higher than that of the other parameters. Although the ROC value was lower than that of the SOFA score, there was no statistically significant difference, suggesting consistent accuracy in predicting both short-term and long-term outcomes. Detailed results are presented in **Table 3** and **Fig 3**.

**Table 3:** Information of ROC curves in Fig 3.

Variable	AUC(%)	95%CI(%)	Threshold	Sensitivity	Specificity
**7-d**					
TBAR	0.650	0.513 - 0.787	1.333	0.542	0.826
Total.Bilirubin	0.631	0.494 - 0.768	3.300	0.542	0.773
Albumin	0.580	0.448 - 0.713	2.500	0.500	0.757
SOFA	0.737	0.623 - 0.850	3.000	0.750	0.695
GCS	0.474	0.387 - 0.561	14.000	0.250	0.814
**14-d**					
TBAR	0.658	0.564 - 0.753	1.333	0.476	0.834
Total.Bilirubin	0.628	0.527 - 0.729	7.000	0.333	0.924
Albumin	0.604	0.510 - 0.698	2.500	0.429	0.761
SOFA	0.671	0.584 - 0.758	3.000	0.571	0.696
GCS	0.458	0.388 - 0.529	14.000	0.262	0.818
**21-d**					
TBAR	0.656	0.571 - 0.740	1.333	0.444	0.839
Total.Bilirubin	0.623	0.535 - 0.711	2.100	0.556	0.678
Albumin	0.610	0.524 - 0.697	2.500	0.463	0.771
SOFA	0.676	0.598 - 0.754	5.000	0.389	0.884
GCS	0.446	0.381 - 0.511	14.000	0.278	0.828
**28-d**					
TBAR	0.641	0.562 - 0.721	1.333	0.413	0.841
Total.Bilirubin	0.617	0.537 - 0.698	2.100	0.540	0.681
Albumin	0.578	0.495 - 0.661	2.500	0.413	0.768
SOFA	0.696	0.624 - 0.767	5.000	0.413	0.893
GCS	0.460	0.401 - 0.518	14.000	0.254	0.821
**90-d**					
TBAR	0.602	0.535 - 0.668	0.757	0.469	0.709
Total.Bilirubin	0.580	0.512 - 0.648	2.1	0.490	0.688
Albumin	0.571	0.501 - 0.640	2.500	0.396	0.780
SOFA	0.677	0.614 - 0.739	2.000	0.646	0.645
GCS	0.465	0.417 - 0.513	14.000	0.240	0.824
**365-d**					
TBAR	0.604	0.544 - 0.664	1.263	0.339	0.830
Total.Bilirubin	0.590	0.529 - 0.651	2.200	0.458	0.702
Albumin	0.547	0.483 - 0.611	2.500	0.356	0.777
SOFA	0.630	0.570 - 0.690	5.000	0.314	0.908
GCS	0.483	0.440 - 0.526	11.000	0.059	0.975

ROC, receiver operating characteristic; AUC, area under the curve; CI, confidence interval; TBAR, Total.Bilirubin to Albumin Ratio; SOFA, Sepsis-related Organ Failure Assessment score; GCS, Glasgow Coma Scale;

### 3.4 Restricted cubic spline analysis of TBAR in relation to AP prognosis

**Fig 4:**
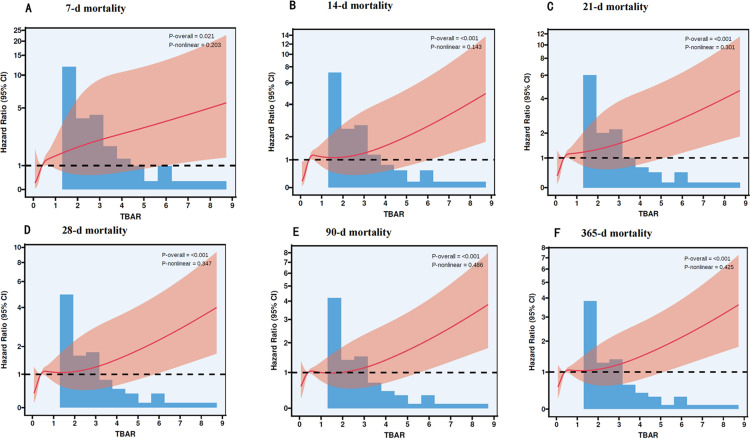
Association between TBAR and Survival with the RCS function at 7-d (A), 14-d (B), 21-d (C), 28-d (D), 90-d (E), and 365-d (F) after admission.

In this study, we utilized RCS analysis to examine the relationship between TBAR and mortality rates at specific time intervals following baseline assessment. The results demonstrated a significant linear association between TBAR and mortality, which was consistent across various intervals. These intervals included 7 days (P = 0.021, P-Nonlinear = 0.203), 14 days (P < 0.001, P-Nonlinear = 0.143), 21 days (P < 0.001, P-Nonlinear = 0.301), 28 days (P < 0.001, P-Nonlinear = 0.347), 90 days (P < 0.001, P-Nonlinear = 0.486), and 365 days (P < 0.001, P-Nonlinear = 0.486). At each examined time point, TBAR was significantly associated with mortality, suggesting that elevated TBAR levels are linked to a higher risk of adverse clinical outcomes. For detailed results, refer to **Fig 4**.

### 3.5 Subgroup analyses of TBAR in AP patients

**Fig 5:**
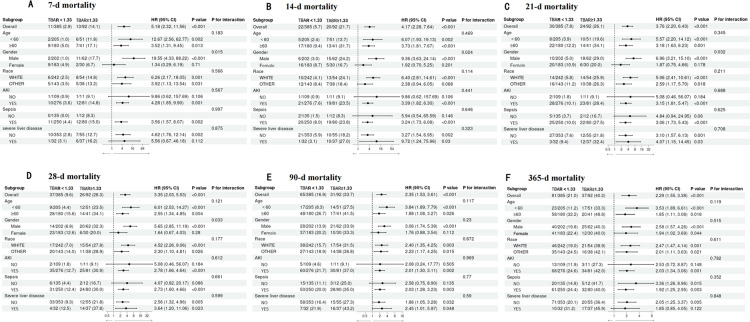
Forest plots of subgroup analysis of the relationship between all-cause mortality and TBAR in patients with AP admitted 7-d (A), 14-d (B), 21-d (C), 28-d (D), 90-d (E), and 365-d (F).

In our subgroup analyses, we evaluated the influence of several factors—age, race, AKI, sepsis, and severe liver disease—on outcomes. No significant interactions were observed between these factors and TBAR. Notably, in the gender subgroup analysis, significant interactions were detected for early mortality rates at 7, 14, 21, and 28 days. However, these interactions attenuated over time and became non-significant at 90 and 365 days. Detailed results are presented in **Fig 5**.

## 4 Discussion

Our study demonstrated that the TBAR is an independent predictor of short-term and long-term all-cause mortality in patients with AP, as confirmed by both univariate and multivariate analyses. KM survival analysis revealed that patients with a TBAR ≥ 1.33 had significantly worse short-term and long-term prognoses compared to those with a TBAR < 1.33 (P < 0.001). In the context of long-term mortality prediction, Although the ROC value was lower than that of the SOFA score, there was no statistically significant difference, thereby establishing TBAR as a reliable prognostic indicator. Additionally, RCS analysis indicated a linear relationship between increasing TBAR values and mortality rates among AP patients. Subgroup analyses further validated TBAR’s accuracy in predicting clinical outcomes in AP patients. Gender differences were observed, which may be attributed to the influence of estrogen. Previous studies have shown that estrogen enhances bilirubin metabolism by regulating cytochrome P4502A6[13]. Moreover, estradiol has been shown to upregulate heme oxygenase - 1 (HO - 1) expression and activity, leading to increased carbon monoxide production. This, in turn, helps maintain microcirculation and mitigate hepatic cell dysfunction and injury [14,15]. Estrogen may also reduce bilirubin levels by influencing the activity of UDP - glucuronosyltransferase in the liver [16]. Collectively, these actions exert a positive influence on bilirubin metabolism. Conversely, estrogen downregulates the expression of adhesion and chemokine molecules in response to inflammatory stimuli, thereby diminishing the recruitment and adhesion of leukocytes to the endothelium induced by inflammatory agonists [17]. This anti - inflammatory effect can indirectly impact albumin levels. Regarding etiology, a large - scale global disease burden study revealed significant gender - based differences in the causes of acute pancreatitis. Men are predominantly affected by alcohol - related pancreatitis, which is more likely to result in liver damage [18]. Impaired hepatic bilirubin metabolism consequently leads to bilirubin accumulation and elevated levels in the body. Sachit et al. [19] conducted a large - scale retrospective population study and found that men with acute pancreatitis had a significantly higher mortality rate than women (P < 0.05). This may lead to lower albumin levels and higher bilirubin levels in men. The protective and anti - inflammatory effects of estrogen may exacerbate the albumin level differences between genders. Over time, however, complications and treatment may gradually weaken this interaction. This explains the observed gender interaction in short - term subgroup analyses, which became less pronounced over time and disappeared in long - term follow - up exceeding 90 days.

In the prognostic evaluation of AP, multiple scoring systems have been developed to assess disease severity. However, these systems often involve complex criteria that limit their utility in clinical practice for diagnosis and treatment. In recent years, ratio-based indicators have gained considerable attention for their ability to predict disease severity and outcomes effectively. Studies have identified several key ratios, including the neutrophil-to-lymphocyte ratio [20], red cell distribution width-to-platelet ratio [21], blood glucose-to-lymphocyte ratio [22], and lactate-to-albumin ratio [23], as significant predictors of AP prognosis. These indicators are easily accessible and cost-effective, providing clinicians with a simple and efficient means for rapid prognostic assessment.

The TBAR has been widely investigated as a prognostic indicator in various diseases. Zhang et al. [24] conducted a retrospective study of 188 patients with ampullary adenocarcinoma and demonstrated that TBAR is an independent prognostic factor, using preoperative TBAR scores and survival analysis. Huang et al. [25] performed a retrospective analysis of 3,442 patients with acute kidney injury, revealing a mortality rate of 25% in the high-TBAR group. After multivariate adjustment, the HR for the high-TBAR group compared to the low-TBAR group was 1.15 (95% CI: 1.03–1.28), further confirming TBAR as an independent risk factor for acute kidney injury. In a study involving 404 patients with traumatic brain injury, Bai et al. reported that the HR adjusted for multiple factors, including the bilirubin-to-albumin ratio, was 1.217. This finding suggests a significant association with the prognostic risk factors for patients [26]. Additionally, studies [27] have shown that bilirubin encephalopathy is closely associated with TBAR, with higher TBAR levels increasing the risk of bilirubin encephalopathy by 23% (OR = 1.23, 95% CI: 1.16–2.48). In a study of 204 patients with liver failure, Li et al. [9] demonstrated that the indirect bilirubin to albumin ratio remains an independent prognostic factor for hepatic encephalopathy, even after univariate and multivariate adjustments (HR = 1.62, 95% CI: 1.32–2.00). Collectively, these studies underscore the prognostic value of the bilirubin to albumin ratio across different diseases, providing a robust theoretical foundation for our research.

Elevated total bilirubin levels, representing the end product of bilirubin metabolism, are commonly associated with pathological conditions such as liver dysfunction and biliary obstruction [28]. In patients with pancreatitis, increased total bilirubin levels may result from biliary obstruction or impaired liver function secondary to pancreatic inflammation [29,30]. Hyperbilirubinemia is not merely a marker of pancreatitis severity but can also exacerbate pancreatic tissue damage through the induction of oxidative stress and inflammatory responses [31]. Additionally, elevated bilirubin levels may disrupt the gut microbiota via altered bile acid metabolism, thereby worsening systemic inflammation [32]. Popa et al. [33] identified an 82.2% correlation between total bilirubin levels and mortality in a retrospective study involving 238 patients with pancreatitis, which may be attributable to liver dysfunction. Similarly, Yu et al. [34] demonstrated that total bilirubin is an independent risk factor for predicting in-hospital mortality in acute pancreatitis (OR=1.247) and has been incorporated into a combined predictive model. A retrospective study focusing on the prognosis of elderly patients with acute pancreatitis also confirmed that bilirubin is an independent risk factor for prognosis [35]. Consequently, elevated total bilirubin levels are significantly associated with adverse outcomes in patients with pancreatitis [36], underscoring its utility as a prognostic indicator. Albumin, the primary plasma protein synthesized by the liver, plays a crucial role in maintaining plasma colloid osmotic pressure, transporting various substances, and providing anti-inflammatory and antioxidant effects [37]. Hypoalbuminemia is frequently observed in severe cases of pancreatitis and may result from decreased hepatic synthesis due to inflammation, increased protein catabolism, and capillary leakage [38]. Reduced albumin levels can further exacerbate pancreatic tissue damage and systemic inflammation by diminishing its anti-inflammatory and antioxidant functions [39,40]. Hypoalbuminemia not only reflects the patient’s nutritional status and inflammation level but is also closely associated with the incidence of complications and mortality in pancreatitis [41].

The correlation between total bilirubin and albumin levels with pancreatitis prognosis underscores the interplay between liver function, nutritional status, and the pathological processes of pancreatitis. Systemic inflammation and oxidative stress, triggered by pancreatitis, are the primary drivers of impaired liver function and reduced albumin synthesis [42,43]. Biliary obstruction and cholestasis further strain liver function, leading to elevated total bilirubin levels [44]. These pathological changes interact, forming a vicious cycle that ultimately deteriorates the prognosis in pancreatitis patients.

The TBAR serves as a composite indicator that integrates these two markers into a ratio, leveraging their inverse relationship to provide a comprehensive assessment of disease severity. This ratio simultaneously reflects liver function, inflammatory status, and oxidative stress levels, making it a valuable tool for prognostic evaluation in clinical practice. By integrating two readily accessible laboratory parameters into a single, interpretable metric, the TBAR provides significant clinical utility, particularly in emergency settings involving critically ill patients with pancreatitis. The straightforward calculation of TBAR allows for rapid identification of patients at higher risk of adverse outcomes. Elevated TBAR values typically reflect increased total bilirubin levels and decreased albumin levels, prompting clinicians to adopt more aggressive treatment strategies, such as early fluid resuscitation and enhanced nutritional support. Additionally, TBAR facilitates risk stratification and improves patient outcomes. Overall, TBAR serves as a simple yet effective prognostic marker, offering reliable predictive value for the severity of AP and patient prognosis.

Biliary and alcoholic pancreatitis are widely acknowledged as the primary etiological factors of AP [37]. Biliary pancreatitis predominantly arises from biliary obstruction triggered by gallstones, which is intrinsically linked to the elevation of bilirubin levels [45]. Conversely, The development of hyperbilirubinemia in alcoholic pancreatitis is primarily driven by hepatic injury and inflammatory cascades [46,47]. Prolonged alcohol consumption in individuals with alcoholic pancreatitis exacerbates hypoalbuminemia, attributable to hepatic dysfunction and chronic malnutrition [48]. Furthermore, the severity of acute pancreatitis may act as a potential confounding factor and exert a certain influence on the study outcomes. Severe cases frequently manifest multi-organ dysfunction, predisposing patients to marked elevations in bilirubin levels, pronounced fluid redistribution, and hypoalbuminemia secondary to malnutrition [49]. Consequently, the TBAR values in severe pancreatitis are significantly higher compared to those in mild cases. The two confounding factors mentioned above may lead to variability in the predictive performance of TBAR.

This study employed a public database to analyze the prognostic factors of diseases. Despite the data being sourced from real-world scenarios, the study is subject to several limitations. First, as a single-center investigation, the study may be biased by geographical, demographic, or healthcare setting differences, which limit the generalizability of the findings. Second, certain key variables, such as etiological information, disease severity stratification, and data for non - ICU patients, were severely missing and unavailable due to database limitations, thereby hindering in-depth analyses of specific issues. Finally, the study population spans a long period, which may not fully account for the impact of contemporary medical advancements on mortality rates.

## 5 Conclusion

Our study indicates that the TBAR effectively predicts mortality outcomes in patients with pancreatitis, both in the short and long term. This finding supports the potential utility of TBAR as a prognostic marker for AP. However, these results require validation through further research.

## Supporting information

S1 File Data. (XLSX)
